# Comorbidities and prescribed medications in expectant mothers attending antenatal clinic: a cross-sectional study in Windhoek, Namibia

**DOI:** 10.1017/S1463423625000350

**Published:** 2025-05-16

**Authors:** Bonifasius Siyuka Singu, Magdalena Maketo, Martha Siwombe

**Affiliations:** School of Pharmacy, Faculty of Health Sciences & Veterinary Medicine, University of Namibia, Windhoek, Namibia

**Keywords:** Anaemia, antenatal, hypertension, Namibia, pregnancy

## Abstract

**Aim:**

The purpose of this study was to report on the prevalence of hypertension and anaemia, and types of medications prescribed to expectant mothers attending antenatal clinics at Intermediate Hospital Katutura in Windhoek, Namibia.

**Background:**

Millennium Development Goals 4 and 5 speak to reduction of child mortality and improvement of maternal health by 2015, respectively. Gestational hypertension is a major contributor to maternal and perinatal mortality and is reported to affect up to 10% of women world-wide. Prevalence of anaemia among pregnant women is reported higher in low- and middle-income countries than in developed countries.

**Methods:**

This was a cross-sectional study involving the review of outpatient and clinic health records for patients attending antenatal clinics at Intermediate Hospital Katutura, Windhoek during October to November 2022. Data for patients on first antenatal clinic visit were obtained from facility antenatal clinic patient registers while that of follow-up patients were from patient health passports. All expectant mothers over 18 years of age who had provided written consent to participate, were included. Data collected were: age, body weight, haemoglobin concentration, blood pressure, gravida, number of babies delivered, pregnancy stage, comorbidities, and prescribed medications. The results were summarised using descriptive statistics. A p-value <0.05 is considered to be statistically significant.

**Findings:**

354 records were included: 303 (85.6%) first visit, and 51 follow-up (14.4%). There was a significant correlation between systolic blood pressure (BP) and body weight (r = 0.31, p < 0.001). 13.5% of first-time visitors had haemoglobin levels lower than the normal range (11 g/dL). Difference in haemoglobin levels between trimesters 1 and 3 were significant (p < 0.001). Methyldopa was prescribed for all hypertensive expectant mothers. To reduce the incidences of anaemia and hypertension during pregnancy, women of childbearing age should be encouraged to attend antenatal visits earlier in pregnancy and to take measures for body weight reduction, respectively.

## Introduction

Millennium Development Goals 4 and 5 speak to reduction of child mortality and improvement of maternal health by 2015, respectively (United Nations, 2015). The Sustainable Development Goals report of 2023 states that 73% of countries globally had made progress in improving child mortality whereas more effort still needed in reducing maternal mortality and improving universal health care, especially in Sub-Saharan Africa (United Nations, 2023). Gestational hypertension is reported to affect up to 10% of women world-wide, and is a major contributor to maternal and perinatal mortality (American College of Obstetricians and Gynecologists, 2019; National Institute for Health and Care Excellence, 2019). The prevalence of anaemia among pregnant women is reported to be as high as 45% in low- and middle-income countries compared to 18–20% in developed countries (Haidar and Pobocik, 2009; Majoni *et al.*, 2021; Kanu *et al.*, 2022; Alem *et al.*, 2023).

In Namibia, efforts have been made by the Ministry of Health and Social Services to reduce the Maternal Mortality Ratio to the global set target of 70/100 000 livebirths by the year 2030 (Heemelaar *et al.*, 2023). However, a surveillance recommends improvements in local antenatal care policies and programmes to improve maternal healthcare outcomes (Heemelaar *et al.*, 2020). A national enquiry into maternal deaths in Namibia found that hypertensive disorders were the second leading cause, after obstetric haemorrhage, of maternal deaths occurring from the years 2008–2019 (Heemelaar *et al.*, 2023). Although the prevalence of anaemia in pregnant women is reportedly low in Namibia, it is still an important indicator to assess the quality of maternal health services as iron deficiency anaemia is a risk factor of perinatal complications such as haemorrhage (Mizanur *et al.*, 2016).

In 2013, more than 97% of women in Namibia received antenatal care from a doctor or trained nurse/midwife, with 43% of these expectant mothers making their first visit in their first trimester (Ministry of Health and Social Services, 2014). The Ministry of Health and Social Services recommend for monthly antenatal visits until 28 weeks of gestation, and then every two weeks up to 36 weeks of gestation and then weekly until delivery (Ministry of Health and Social Services, 2011). The percentage of expectant mothers who made at least four visits to the antenatal care clinic as recommended by the World Health Organisation decreased from 70% in 2006 to 63% in 2013 (Ministry of Health and Social Services, 2014; World Health Organization, 2016).

The setting for this study was Intermediate Hospital Katutura which is one of the two largest hospitals in Namibia, based in the capital city Windhoek. The antenatal clinic is staffed with 5 registered nurses, 4 medical doctors, and 2 intern medical doctors. Nurses record patient details in record books and carry out physical examinations such as palpations, body weight and blood pressure measurements, test for haemoglobin, and urine dipstick analysis for glucose. The medical doctors perform sonars, prescribe medications, and manage any other matters referred by nurses. On average, 186 expectant mothers attend the antenatal clinic monthly (2,232 annual visitors).

Windhoek, the largest city in Namibia, is based in Khomas region which reported the highest prevalence of maternal deaths caused by postpartum haemorrhage and hypertensive disorders such as preeclampsia (Ministry of Health and Social Services, 2014). Although the numbers of expectant mothers attending antenatal clinics in Namibia are high and health care services are accessible in most parts of the country, the types of medications prescribed to manage comorbidities in pregnancy has not been reported.

This study set out to answer the following questions: what are the common comorbidities among expectant mothers who attend antenatal clinics at Intermediate Hospital Katutura in Windhoek, Namibia? Furthermore, what medications are prescribed to these women?

## Methods

### Study design and area

This was a cross-sectional study involving the review of 354 outpatient and clinic health records for patients who attended the antenatal clinic at Intermediate Hospital Katutura, a major state hospital in Windhoek, Namibia. A cross-sectional study was well-suited to answer the question of prevalence and because time was not sufficient to conduct a longitudinal study to follow the same expectant mothers from first visit all through their pregnancy.

### Study population and sampling

Only patients who had their first antenatal clinic visit from October to November 2022 were included, and their data were obtained from antenatal clinic patient registers kept at the facility. Data for follow-up patient were collected from patient health passports which are kept by patients. Only expectant mothers who had their outpatient medical record book or health passports at the time of being approached for consent to participate were included in this study. Patients who were younger than 18 years and those who did not consent to taking part in the study were excluded. Informed written consent was obtained from follow-up patients at the antenatal clinic on Mondays and Thursdays before accessing their health passports.

### Data collection procedure

Data were collected from patient health passports immediately after written informed consent was obtained. The following data were collected from the clinic records book at as detailed at the first visit: patient age, body weight, haemoglobin concentration, blood pressure, gravida (number of pregnancies), para (number of babies delivered), pregnancy stage, blood pressure, initially prescribed medications (ferrous fumarate + folic acid, and multivitamins). Furthermore, data such as age, trimester, comorbidities, and other prescribed medications were also collected from the patient health passport.

### Ethical considerations

Approval to conduct this study was granted by the Research and Ethics Committee of the Ministry of Health and Social Services, Republic of Namibia (Ref: 22/3/1/2). Written permission was obtained from the office of the medical superintend at Intermediate Hospital Katutura before commencing the study at the antenatal clinic, while informed written consent was obtained from individuals who participated. To ensure patient confidentiality, patient names were not collected as part of this study. Additionally, this data was only accessible to researchers.

### Data analysis

In the present study, hypertension in pregnancy was defined as a systolic blood pressure measurement >140 mmHg with a diastolic of >90mmHg (National Institute for Health and Care Excellence, 2021; Goławski *et al.*, 2023). Anaemia in pregnancy was defined as haemoglobin levels of < 11 g/dL (World Health Organization, no date; Breymann *et al.*, 2017; Pavord *et al.*, 2020). The results were summarised using descriptive statistics in R (version 3.6.2) and Excel (Microsoft Corporation Redmond, Washington). The Mann-Whitney U test was used to compare medians, with a p-value <0.05 considered to be statistically significant.

### Reflexivity

Because this study had a brief data collection window period of only two months, there was not enough time to conduct a more suitable study that could follow the same cohort of expectant mothers from the first visit all through to their third trimester.

## Results

### Patient demographics

A total of 354 patients were included in the study, of which 303 (85.6%) were first-time visitors, and 51 (14.4%) were follow-up attendees. As this was a cross-sectional study, the 51 follow-up patients were not necessarily part of the 303 included as first-time visitors. For the expectant mothers that came in for the first visit, 38 (12.5%) were in their first trimester, 184 (60.7%) were in their second trimester, and 81 (26.7%) were in their third trimester. Age and weight ranges of all 303 first-visit expectant mothers were 17–46 years and 36-181 kg, respectively. Patient demographics are shown in Table 1. Among the women who came for their first antenatal visit during the study period, 15.5% (age range: 17–38 years) were on their first pregnancy. Furthermore, 46/303 of first-time visitors (15.2%) had at least one previous pregnancy which did not come to term, with age groups 36–46, 26–35, and 17–25 years contributing 37.0%, 47.8%, and 15.2%, respectively.

**Table 1.** Summary of patient demographics at first visit (303)

Characteristics	Median (range)
Age (years)	30 (17–46)
Pregnancy stage, n (%) First trimester. 38 (12.5) Second trimester. 184 (60.7) Third trimester. 81 (26.7)	2 (1–3)
Number of pregnancies (gravida)	3 (1–11)
Number of successful births (para)	2 (0–10)
Gestation (weeks)	22 (3–40)
Systolic BP (mm Hg)	122 (91–186)
Diastolic BP (mm Hg)	77 (54–139)
Haemoglobin (g/dL)	12.3 (6.5–15.1)
Weight (kg)	68 (36–181)

### Findings on blood pressure

**Figure 1.**
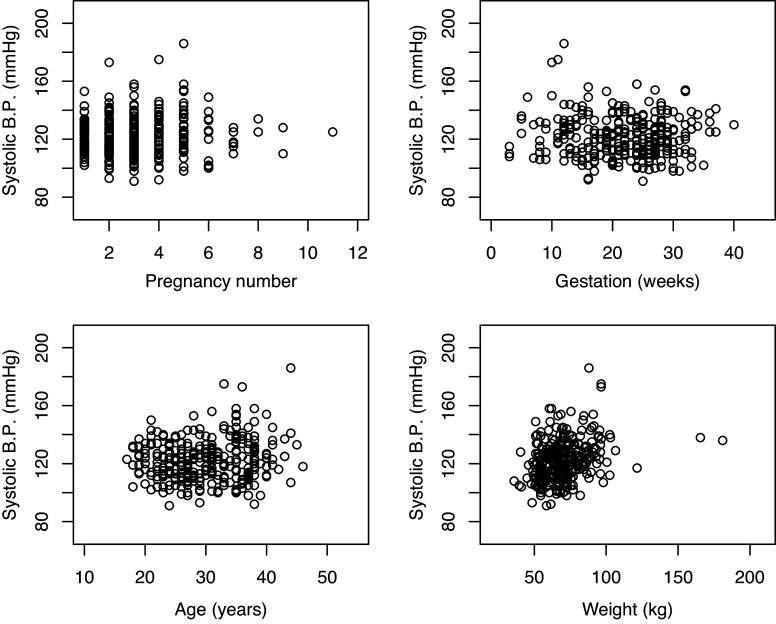
Relationship of various demographics with systolic blood pressure. Systolic blood pressure has a positive association with body weight of expectant mothers, r=0.311 and p < 0.001 (n = 303).

**Figure 2.**
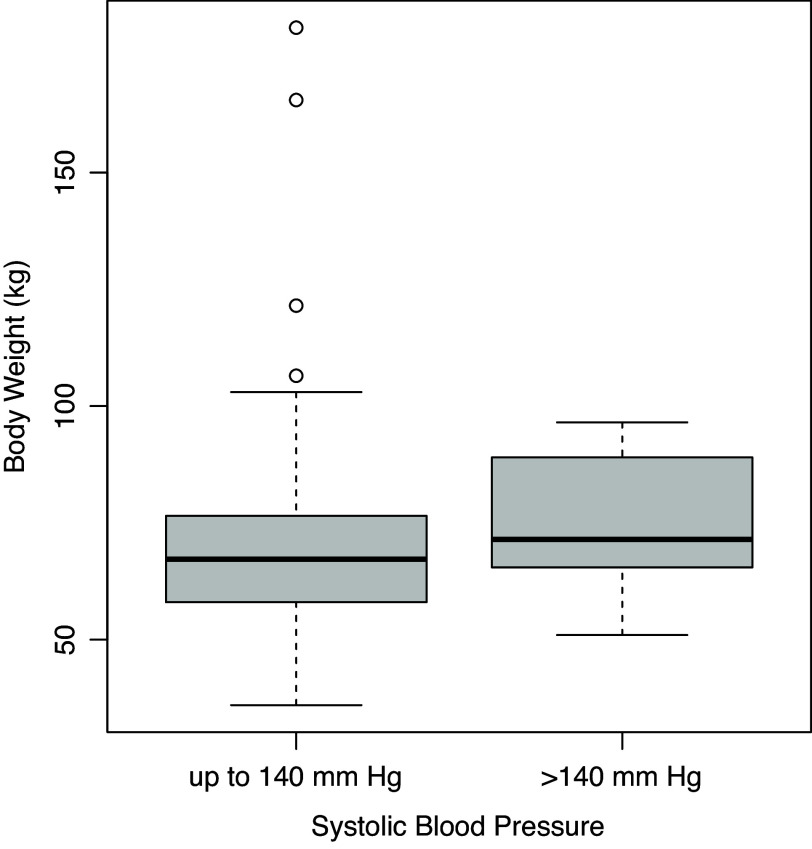
Expectant mothers with BP outside the normal range (n = 26) had statistically higher body weight (p = 0.017) than those with normal BP (n = 277).

At the first visit, 38 (12.5%) of expectant mothers had systolic blood pressure levels that were over 140 mmHg. There was no statistically significant correlation between systolic blood pressure (BP) and length of gestation (trimester), pregnancy number, nor age of expectant mothers (Figure 1). However, there was a positive and statistically significant correlation between systolic blood pressure and body weight (r=0.31, p<0.001). Furthermore, expectant mothers who had a high blood pressure were of a body weight that was significantly higher (p = 0.017) than those with BP in the normal range (Figure 2).

### *Findings on* haemoglobin

**Figure 3.**
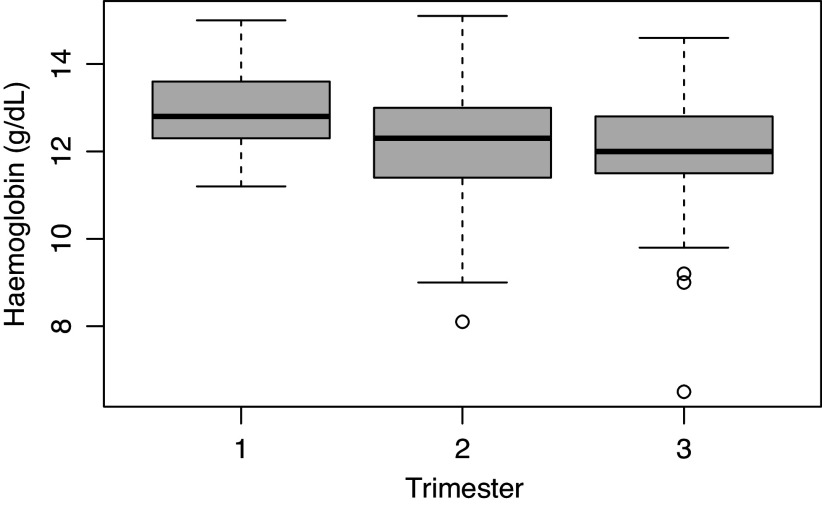
Haemoglobin levels across semesters (n = 303).

41 (13.5%) of patients in this study had haemoglobin levels that were less than the lower limit of the normal range (11 g/dL) at their first visit. The difference between haemoglobin levels was statistically significant for patients in trimesters 1 and 2 (p = 0.001), and trimesters 1 and 3 (p < 0.001), but not significant between trimesters 2 and 3 (p = 0.23), see Figure 3.

### Prescribed medications

Most of the patients (64.7%) received 1-2 prescribed medications during follow-up. The 18–25 years old age group (n = 16) received an average number of 2.4 medicines, while the 26-35 years (n = 24) received 2.6 medicines. The 36–45-year-old age group (n = 11) had the most prescribed medicines at 3.8. Virtually all patients received Ferrous fumarate + folic acid and multivitamins (Table 2). Patients with hypertension (n = 8, 15.7%) were all managed with methyldopa (Table 2).

**Table 2.** Summary of parameters during follow-up (n = 51)

Age (years), median (range)	29 (18–45)
Pregnancy stage, n (%)	
First trimester	0 (0)
Second trimester	9 (17.6)
Third trimester	42 (82.4)
Median (range)	3(2-3)
Comorbidities, n (%)	
Hypertension	8 (15.7)
Diabetes	1 (1.95)
Vaginal discharge syndrome	3 (5.9)
HIV/AIDS	1 (1.95)
None	38 (74.5)
Number of drugs, n (%)	
1-2	33 (64.7)
3-4	9 (17.6)
5+	9 (17.6)
Most frequently prescribed drugs, n (%)	
Ferrous fumarate + folic acid	51 (100)
Multivitamins	50 (98)
Aspirin	8 (15.7)
Methyldopa	8 (15.7)
Calcium gluconate	7 (13.7)
Paracetamol	4 (7.8)
Metronidazole	3 (5.9)
Azithromycin	2 (3.9)
Cefixime	2 (3.9)
Clotrimazole	2 (3.9)
Metoclopramide	1 (2.0)
Metformin	1 (2.0)
Pyridoxine	1 (2.0)
TDF/3TC/DTG	1 (2.0)

## Discussion

Although antenatal care has been accepted by virtually all World Health Organisation member states as an important primary healthcare measure to safeguard maternal and child health, many countries in Sub-Saharan Africa are still lagging behind in set targets (United Nations, 2015, 2023).

In this study, a small number (12.5%) of expectant mothers attended their first antenatal visit during the first trimester of their pregnancy, a figure which is much lower than 44% reported in an earlier national survey (Ministry of Health and Social Services, 2014). This should be a concern as it does not signify improvement of maternal and child health care. Although it is not clear what the reasons were for poor attendance of antenatal clinics during the first trimester, a survey carried out earlier in Namibia, and corroborated by other studies such as the one in Malawi, indicate common causes to be socioeconomic pressures, beliefs, and attitudes, such as: a lack of transportation, waiting to miss several periods before confirming a pregnancy, distance to health facility, unhelpful/rude clinic staff, and payment of treatment fees (Ministry of Health and Social Services, 2000; Finlayson and Downe, 2013; Chimatiro *et al.*, 2018). A study in Ghana reports that maternal age, level of education, and household socioeconomic status are factors that affect when expectant mothers make their first antenatal care visit (Manyeh *et al.*, 2020). Another study conducted in Peru reports determinants of first-trimester antenatal care attendance as the following: living with a partner, completion of secondary schooling, residence in an urban area, and history of a miscarriage (Moore *et al.*, 2017). Although interventions have proven effective in improving the frequency of attendance, early initiation has remained a challenge; a study in Uganda to assess the effect of community health workers to promote antenatal services proved ineffective in improving first-time visits during the first trimester and recommends for other approaches of promotion of early initiation to be explored (Wafula *et al.*, 2022). This study only captured clinical data and left out socioeconomic demographics which would have been useful in shedding some light as to why the vast majority of the study participants did not attend their first antenatal visit in the first trimester. Nonetheless, the observed delay in initial attendance requires interventional efforts in order to improve maternal and child health which in this study would be reduction of prevalence of hypertension and anaemia. However, if the reasons mentioned earlier as to why expectant mothers delay the first visit are the same for participants in this study (Ministry of Health and Social Services, 2000; Finlayson and Downe, 2013; Chimatiro *et al.*, 2018), then the possible solutions could be: community-based education on the importance of early visitation of antenatal clinic, involvement of the male partner, education of clinic staff on professional patient care practices to encourage attendance at the clinic, and governmental efforts to bring healthcare services closer to the communities, especially in rural areas (Totade *et al.*, 2023). Interventions at government level to improve early antenatal visits could include provision of mobile clinics and increasing the number of clinics per population.

Hypertension was found to be the most common co-morbidity with a prevalence of 15.7%. In this study, systolic blood pressure of expectant mothers was found to be associated with their body weight, but not with other variables such as age, number of pregnancies, or stage of gestation. Being overweight, obese, and excessive gestational weight gain have been reported to be associated with development of gestational diabetes mellitus and gestational hypertension (Sun *et al.*, 2020; Madlala *et al.*, 2023; Lackovic *et al.*, 2024). Furthermore, the odds for developing hypertension and preeclampsia per 10 kg pre-pregnancy weight have been found to be higher for expectant mothers with a body weight higher than 90 kg when compared to those with a weight of 90 kg or less before pregnancy (Macdonald-Wallis *et al.*, 2013). Apart from hypertension, excessive gestational weight gain has been shown to be associated with other adverse pregnancy outcomes such as higher birthweight, caesarean section, hospitalisation, and a low Apgar score (Goławski *et al.*, 2023). All hypertensive expectant mothers in this study received oral methyldopa 250 mg daily as pharmacotherapy, which is the Namibia Standard Treatment Guidelines recommended first-line drug (and lowest dose) for maintenance therapy in pregnancy and in line with international guidelines (Ministry of Health and Social Services, 2021; Michel and Hoffman, 2018; National Institute for Health and Care Excellence, 2023). However, in order to mitigate the risk of maternal or infant morbidity it is pertinent for expectant mothers to aim for a normal body-mass index through healthy diet and exercise, after all: “prevention is better than cure.” Earlier attendance of first visit could be instrumental in expectant mothers receiving the necessary information on the importance of a healthy diet and exercise in prevention of gestational hypertension rather than having a delayed first visit when the pregnancy has advanced. Limiting perinatal weight gain with physical exercise and healthful diet are inexpensive interventions which are readily available and safe but must be applied earlier on in the pregnancy (Health, 2023). Body-mass index was not part of the variables recorded in either the health passport carried by the expectant mothers nor in the records book which is permanently kept by the antenatal clinic. This is a drawback in the quality of data capturing at this facility as body-mass index is a useful variable in monitoring weight changes for any patient.

A notable proportion (13.5%) of expectant mothers had anaemia at the first visit, with levels of haemoglobin becoming significantly with increase in gestational age. The most common type of anaemia during pregnancy is iron deficiency anaemia, followed by folate deficiency megaloblastic anaemia which is mainly due to poor iron diet or a lack of iron and folate supplementation during the antenatal period (Sifakis and Pharmakides, 2006). The Namibian Standard Treatment Guidelines recommend ferrous fumarate and folic acid supplements for the duration of pregnancy to prevent anaemia (Ministry of Health and Social Services, 2021). In low- and middle-income countries, common factors associated with anaemia include: level of education, socioeconomic status, family size, exposure to media, and residence (Alem *et al.*, 2023). A study in India recommends promotion of frequent antenatal visits, iron supplements, consumption of iron-fortified foods, and offering pregnant women with financial assistance in order to reduce maternal mortality caused by gestational anaemia (Totade *et al.*, 2023). It is worthwhile to note that all expectant mothers who attended the antenatal clinic at the study site received ferrous fumarate in combination with folic acid supplementation. This speaks to the evidence-based antenatal care that is practised at the facility and demonstrates the intention of the health ministry to offer quality healthcare to expectant mothers who present to receive antenatal services. Although the median haemoglobin was within the reference range, a substantial number were anaemic. In addition, it is noteworthy that the levels decreased with each subsequent trimester for first-time visitors which implies that expectant mothers who present later in pregnancy for antenatal care are at a higher risk of anaemia than those who attend earlier, which once again emphasises the importance of early first antenatal visitation. It would have been interesting had the information on haemoglobin levels been recorded in the patient passport as well. This would have allowed a comparison of haemoglobin concentrations at the first visit as opposed to several months later.

### Study limitations

One limitation of this study is that data were collected at only one clinic in Windhoek instead of all antenatal clinics in the city. Therefore, the results from this study may not be generalisable to the entire Namibian population. Furthermore, the records for the initial visit versus those for the follow-up visits were not from the same patients, the time was not sufficient for a longitudinal study that would have followed the same expectant mothers from first visit to their third trimester. Consequently, the cross-sectional study design was unable to differentiate between different types of hypertensive disorders in these expectant mothers (for example, chronic hypertension vs gestational hypertension). To overcome these limitations, the study included 354 participants which well exceeded the pre-calculated sample size of 339 for statistical significance. In addition, the results were simply discussed from the perspective of how current body weight correlates with the observed blood pressure. The relationship between chronic or gestational hypertension with weight gain in pregnancy could be an interesting topic for future studies.

## Conclusions

The results from this study suggest expectant mothers do not start attending antenatal clinic early enough as very few (12.5%) had their initial visit in the first trimester of their pregnancy. The vast majority (87.4%) were in the second and third trimester at their first visit. 12.5% and 13.5% of pregnant women had hypertension and anaemia at the first visit, respectively. High body weight has a notable influence on diagnosis of hypertension in expectant mothers. All patients (with or without anaemia) received ferrous fumarate and folic acid supplementation while those with hypertension received methyldopa as recommended by the standard treatment guidelines. To reduce the incidences of anaemia and hypertension during pregnancy, government policymakers should find ways to encourage women of childbearing age to attend antenatal visits earlier in their pregnancy and to take measures for body weight reduction, respectively.
